# Feeding influences the oxidative stability of poultry meat treated with ozone

**DOI:** 10.5713/ajas.18.0520

**Published:** 2018-10-26

**Authors:** Andrea Ianni, Lisa Grotta, Giuseppe Martino

**Affiliations:** 1 Faculty of Bioscience and Technology for Food, Agriculture and Environment, University of Teramo, Teramo 64100, Italy

**Keywords:** Ozone, Chicken Meat, Oxidation, Fatty Acids, Water Holding Capacity

## Abstract

**Objective:**

Ozone is considered a strong antimicrobial agent with numerous potential applications in the food industry. However, its high oxidizing potential can induce alterations in foods by acting on the unsaturated fatty acids. The aim of this study was to investigate the effect of ozonation on the oxidative stability of chicken breast meat obtained from animals subjected to different feeding strategies.

**Methods:**

Samples were obtained from commercial hybrid chickens (ROSS 508), some of which were nourished with a feed enriched with fats of animal origin, while the lipid source was vegetal for the remaining birds. Samples of meat belonging to both groups were treated with ozone and then analysis was performed to evaluate alterations in physical properties, lipid content, fatty acid profile, and oxidation stability.

**Results:**

Ozone induced a significant reduction in drip loss in meat samples obtained from animals nourished with vegetable fats; this nutritional strategy also produced meats leaner and richer in polyunsaturated fatty acids. Thiobarbituric acid reactive substances, useful for the assessment of lipid oxidation, were higher in samples obtained from animals fed with vegetable fats with respect to diet based on the addition of animal fats.

**Conclusion:**

The ozone treatment improved the physical parameters of meat samples obtained from animals fed with vegetable fats, however the same samples showed a higher lipid oxidation compared to what observed in the case of the dietary intake of animal fats, probably as a consequence of the marked increase in polyunsaturated fatty acids which are more susceptible to peroxidation.

## INTRODUCTION

Fresh poultry meats are widely consumed because of the low fat and high protein content and the presence of key nutrients such as vitamins and minerals [1]. In all phases of the industrial production microbial contamination represents the main cause of food poisoning, with high risks for consumers, who can face a broad range of pathological events. Although there are different decontaminating methods to control the growth of microorganisms in food and keep the integrity of chemical composition, safety is still the key objective in the food industry. Among existing processes, ozone treatment largely contributes to the improvement of quality and safety of food products. The use of ozone as decontaminating agent, instead of traditional agents such as chloride, is justified by its significant oxidative properties which guarantee a broad spectrum of bactericidal effects [2]. In aqueous systems, ozone can react with chemical compounds through two different but coexisting modes, one involving the direct action of molecular ozone and the other based on free radical-mediated mechanisms. In addition to this, the molecule decomposes spontaneously to oxygen, for that reason the use of ozone in food industry minimizes the accumulation of inorganic compounds in the environment and especially on food surfaces, limiting potential risks for human health [3]. Consequently, the action of ozone should influence also the oxidative stability of macromolecules that make up the food itself, especially the unsaturated fatty acids (UFA) [4].

Several research groups have focused their attention on the level of unsaturation of lipids taken through the diet and their effect on human health [5]. The increase in consumption of saturated fatty acids (SFA), especially in developed countries is related to the increase in deaths due to coronary heart diseases. Compounds such as lauric acid, myristic acid and palmitic acid increase the concentration of total cholesterol in plasma and the concentration of low-density lipoproteins associated with cholesterol (LDL-C), presumably by decreasing the activity of LDL receptor and/or increasing LDL-C production [6]. Blood cholesterol levels are significantly lowered with the intake of polyunsatured fat acids (PUFA), which however are particularly susceptible to peroxidation events leading to the formation of compounds harmful to health such as oxysterols and malondialdehyde (MDA) [7]. In recent years, attempts have been made to replace the SFAs in foods with monounsaturated fatty acids (MUFA) by changing the fatty acids concentration in livestock feed thus obtaining greater MUFA concentrations in their products [8,9].

In this study, two different feeding strategies which differ in the source of the lipid component: animal origin in one case and vegetable in the other, were administered to two groups of chickens. Analysis of breast meat samples were performed to evaluate the influence of diet on the fatty acid profile and the effect of the ozonation on the oxidative stability of meat.

## MATERIALS AND METHODS

### Samples collection and ozonation

Samples of breast chicken meat were obtained from animals belonging to the commercial hybrid line ROSS 508 and reared with the conventional intensive system. Before sampling, chickens were divided into two groups and subjected to different feeding strategies: the “standard line” (SL) was nourished with a diet containing cereals, soybean extraction flour and animal fats (tallow and lard), while for the “vegetal line” (VL) was used a standard feed to which vegetable fats (soybean oil) were added (Tables 1, 2).

**Figure 1:**
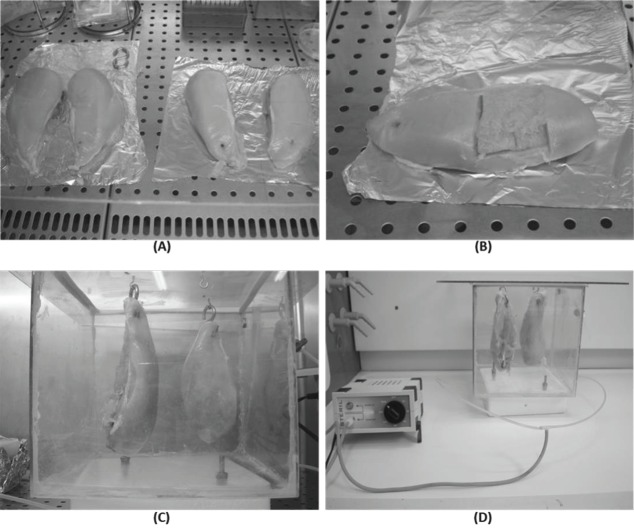
(A) Chicken breasts (8 for each group) were firstly divided in half. (B) In the sampling phase meat aliquots were taken in the surface (0 to 3 mm) and in the inner part (5 to 8 mm). (C) For ozonation pieces of meat were hung through hooks fixed on the upper wall of the chamber. (D) Ozonation was performed at 4°C to 6°C for 5 h using a generator with a production capacity equal to 250 mg/h and a gas volume exchanged by the box equal to 62±0.3 mL/min.

Sixteen chickens (8 for each line), with a live weight between 3.5 and 3.8 kg, were randomly selected and slaughtered in an authorized company, according to current legislation; meat was taken within one hour from the slaughter. Chicken breasts were firstly divided in half (Figure 1A) and one part was immediately treated with ozone. Before the storage both treated and untreated samples from SL and VL were analyzed from a physical point of view, evaluating the colour of the meat surface and drip loss. Aliquots of known weight were obtained at this point and stored at −20°C until use for further investigations. Because we wanted to evaluate the effect of ozone both on the surface and in the innermost part of the meat, samples were taken aliquots in the thickness between 0 and 3 mm and 5 to 8 mm (Figure 1B).

For ozonation, samples were placed in a box with a total volume of 27,000 cm^3^ (dimensions: 30×30×30 cm) and, as is possible to see in Figure 1C, to increase the contact with the gas, the pieces of meat were hung through hooks fixed on the upper wall of the chamber. The total volume occupied by poultry meat in the ozonation chamber ranged between 0.896 and 0.909 cm^3^. Ozonation was performed at 4°C to 6°C for 5 h using a generator (P.M.G. Depurazione, Vercelli, Italy) with a production capacity equal to 250 mg/h (Figure 1D), and a gas volume exchanged by the box equal to 62±0.3 mL/min; the total distance between the generator and the samples was 56 cm, with a bifurcation of the flow 24 cm before entering the box to create 2 access points for the gas and improve its distribution. The ozonation parameters were defined considering the volume of the chamber, the production capacity of the ozone generator and the average time commonly indicated to be useful to obtain a significant reduction of the microbial load on the meat surface [10].

### Drip loss

Samples of fresh chicken breast meat were cut and immediately weighed to obtain pieces of about 70 to 80 g which were placed in a container with a net on the bottom for 24 h at 4°C. Before the final weighing, the surface of each sample was gently dabbed with paper towels as suggested by Correa et al [11]. The drip loss was expressed as a percentage relative to the initial weight.

### Colour evaluation of meat surfaces

The colour analysis was performed on the surface of the chicken breast and on the cut surface free of connective tissue. Measurements were performed with Minolta CR-300 reflectance colorimeter based on the measurement system known as Commission Internationale de l’Eclairage L*a*b* [12]; this system defines the spatial-chromaticity in terms of gloss (L*) and chromatic coordinates a* (redness) and b* (yellowness). The color is determined by the ability of the surface to reflect the different wavelengths of the electromagnetic radiation in the visible spectrum (400 to 700 nm).

### Lipid content determination and evaluation of the acidic profile

The Folch method was used to determine the total lipid amount in meat samples [13]. About 6 g of meat, previously minced and deprived of adipose tissue residues, were weighed and homogenized in test tubes with Ultra Turrax T25, gradually adding 120 mL of Folch solution composed of chloroform (Carlo Erba, Milan, Italy) and methanol (Sigma-Aldrich, Milan, Italy) in a 2:1 ratio. The homogenate was then slow stirred for 6 h at room temperature in a borosilicate flask. Using Whatman filters n°41 (Sigma-Aldrich, Italy), samples were transferred inside separating funnels, where, the addition of 14 mL of 0.9% sodium chloride solution separated the chloroform phase (apolar) containing lipids, and the polar one consisting of water and methanol. After about 12 h the chloroform fraction was filtered and concentrated with a rotary vacuum evaporator at 35°C to 40°C. The lipid extracts were then placed in the oven for 3 h at 70°C and, only after cooling in the dehumidifier, samples were weighed. In this way it was possible to trace the lipid percentage with respect to the total weight of the starting meat sample.

The evaluation of the fatty acid profile in chicken breast meat was performed starting from the total lipid extracts obtained by using the Folch method described above. Each lipid extract was trans-methylated to fatty acyl methyl esters (FAMEs) by adding 2 mL of hexane (Carlo Erba, Italy) and 500 μL of methylating solution (KOH 2 M in anhydrous methanol); FAMEs composition was determined in gas chromatography with flame ionization detection. The chromatograph was a Perkin Elmer AutoSystem XL with the column Varian CP-SIL (88 of 100 meters) and hydrogen as transporter. The thermal program was as follows: 160°C for 3 min, 3°C/min until 175°C for 25 min, 3°C/min until 220°C per 40 min and 10°C/min until 160°C. The identification of fatty acids (FAs) was based on the comparison of retention times of each FA with a standard mixture (Sigma-Aldrich, Italy) and results were expressed as percentage of the sum of total determined FAs.

### Evaluation of lipid oxidation: the thiobarbituric acid reactive substances-test

Fat oxidation was evaluated by measuring thiobarbituric acid reactive substances (TBARS) [14]. The test is based on the reaction of 2 molecules of 2-thiobarbituric acid (TBA) with a molecule of MDA, to form a red pigment with a maximum of absorbance at 534 nm [15]. To optimize the analysis, the procedure described by Grotta et al [16] was followed. For each condition 3.5 g of frozen meat were mixed, within 2 min of sample withdrawal from the freezer, with 500 μL of 0.1% of butylated hydroxytoluene in methanol to stop the oxidation process. Samples were homogenized with Ultra Turrax T25 in 50 mL of an acqueous solution containing 7% trichloroacetic acid, and then subjected to distillation [17]. Each distillate (2 mL) was added with an equal volume of a solution containing 0.02 M TBA in 90% acetic acid. This preparation was kept for one hour in a thermostated bath at 80°C, and only after cooling, the absorbance at 534 nm with a JENWAY 6305 UV/vis spectrophotometer was evaluated. The amount of MDA was calculated for each sample using a calibration curve (R^2^ = 0.99904). Results were expressed in mg of MDA per kg of meat (ppm).

### Statistical analysis

Statistical data processing was performed by the general linear model procedure [18], using the following linear model: y_ijk_ = μ+a_i_+e_i_, where μ = overall average; a_i_ = fixed effect rearing system; e_i_ = error. The significance of differences was estimated by the multiple Student’s t-test. Differences were considered significant for p<0.01 and p<0.05.

## RESULTS AND DISCUSSION

### The ozonation improves physical properties of chicken meat

Table 3 reports the effect of ozonation on the color and water-holding capacity of chicken meats from the different rearing systems. Hughes et al [19] explained in detail the role of water and colour as primary determinant of product quality, visual appearance and sensory appeal [19]. They emphasized the fact that the capacity of meat to retain water is affected by denaturation of myofibrillar proteins with important repercussions on nutritional parameters. Variations in color instead, could be the result of globin denaturation in myoglobin, haem displacement or release and ferrous oxidation; this condition could give rise to a dark colour, influencing consumer acceptability. In our study VL is characterized by significantly brighter meats (L*: 53.20 vs 56.14, SL vs VL respectively, p<0.01), less red (a*: 0.57 vs −1.65, SL vs VL respectively, p<0.05) and less yellow (b*: 17.63 vs 8.53, SL vs VL respectively, p< 0.01). The treatment with ozone had significant effects. In particular, ozonation generated brighter meats (L*: 53.20 vs 54.70 and 56.14 vs 59.28, SL vs ozonized SL (SLO) and VL vs ozonized VL (VLO) respectively, p<0.01) and more red (a*: 0.57 vs 0.97 and −1.65 vs −1.01, SL vs SLO and VL vs VLO respectively, p<0.05). The interaction between the two treatments (food and ozonation) was also significant (p<0.05) on luminosity (L*) and red index (a*).

**Figure 2:**
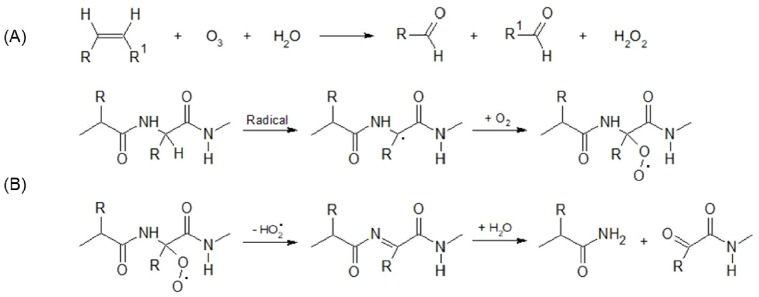
(A) Net reaction of ozonation on an unsaturated site proposed by Criegee [20]. In presence of water the ozone directly interacts with the double bonded C atoms giving origin to ketons or aldehydes while the ozone molecule is reduced to a derivate of hydrogen peroxide. (B) Mechanism described by Davies [23] that could explain protein backbone fragmentation with formation of polar compounds. Chicken breasts (8 for each group) were firstly divided in half.

Regards the drip loss, all effects and their interaction were significant: vegetable based nutrition produced meat with higher dripping losses (0.65% vs 1.49%, SL vs VL respectively; p<0.01), while ozone reduced them (0.65% vs 0.36%, SL vs SLO respectively, p<0.01; 1.49% vs 0.46%, VL vs VLO respectively, p<0.01). Particularly relevant is the fact that ozone treatment limited the drip loss of meat from chickens fed with vegetable fats (p<0.01). The ability of ozonated meat to retain water, probably depends on the presence of polar compounds deriving from ozone-based oxidation processes on PUFAs and proteins. As described by Criegee [20] and Pryor et al [21], in presence of water the ozone directly interacts with the unsaturated sites in PUFAs due to their dipolar nature, giving origin to an “ozonide” that decomposes breaking the fatty acid carbon-carbon bond. Because of this cleavage the two C atoms which were originally double bonded attain the oxidation state of ketons or aldehydes while the ozone molecule is reduced to a derivate of hydrogen peroxide (Figure 2A). In the case of protein (Figure 2B), the oxidation process could be mediated by singlet oxygen which can be produced in high yield by the reaction of ozone with biological molecules [22]. Singlet oxygen can induce the backbone fragmentation with formation of polar compounds through a mechanism, described by Davies [23], that triggers with the formation of backbone radicals via hydrogen atom abstraction from an α-carbon.

### Dietary intake of vegetable fats makes chicken meat richer in PUFAs and more susceptible to peroxidation

Results related to the lipid content and the acidic profile of chicken meat, have highlighted significant differences between the two groups of study. As shown in Table 4, SL is characterized by meat richer in lipids, with a higher concentration of myristic acid (C14:0), palmitoleic acid (C16:1) and total MUFAs, while the VL has produced meat that is significantly richer in stearic acid (C18:0), arachidonic acid (C20:4) and, in general, of total PUFAs. These findings are broadly in agreement with what is reported in the literature, especially as described by Kanakri et al [24], who found that feed based on the use of animal fats reduces the accumulation of omega-6 linoleic and arachidonic fatty acids in chicken meat.

Regarding the development of lipid peroxidation, evalua tions on both the superficial portion (0 to 3 mm) of the meat sample, and the innermost part (5 to 8 mm) show the effects of feeding, the effects of ozonation and their interaction (Table 5). Both conditions induced significant effects (p<0.01, and their interaction p<0.01). The treatment with ozone increased significantly the MDA amount with, as expected, higher levels of oxidation found in samples taken from the surface of the meat, therefore more exposed to the gas. This effect is probably due to the action of the third oxygen atom of the ozone molecule, which is not stably bound and can detach without difficulty, generating a highly reactive oxidizing system that could affect the structural integrity of other compounds. This finding is particularly evident in VL samples, more susceptible to peroxidation due to the higher concentration of PUFAs [25,26]. Oxidation of the PUFA generates fatty acid radical that rapidly adds oxygen forming peroxyl radicals, which can oxidize further PUFAs, producing lipid hydroperoxides that can break down to yet more radical species and to a wide range of compounds, notably aldehydes [27].

## CONCLUSION

Based on the obtained results, it can be stated that dietary intake of vegetal supplements produced meat significantly leaner and richer in PUFAs, but, at the same time, more susceptible to peroxidation, creating a condition which isn’t suitable for ozone treatment [28]. The physical profile the two types of meat showed different properties but, in both cases, the treatment with ozone improved the water retaining capacity, increasing it. These positive aspects, together with the well-known antibacterial activity carried out by ozonation, indicate a need for further research on the oxidative stability of meat obtained from chicken fed with vegetable-based diets, through the addition of soluble antioxidant vitamins [29,30].

## Figures and Tables

**Table 1:** Chemical composition of experimental diets

Diets	Standard line	Vegetal line
Chemical composition (%)		
Crude protein	19.50	19.30
Ether extract	7.50	6.50
Crude fiber	3.00	3.10
Ash	4.50	4.70

Ingredients (presented in decreasing order) were: SL: wheat, soybean meal, sorghum, animal fats, corn, dicalcium phosphate, calcium carbonate, sodium chloride, sodium bicarbonate. VL: wheat, soybean meal, sorghum, soybean oil, corn, dicalcium phosphate, calcium carbonate, sodium chloride, sodium bicarbonate.

**Table 2:** Fatty acid profile of feeds and lipid supplements of animal and vegetable origin

Fatty acids (%)	Feeds		Animal fat	Soybean oil

	Standard line	Vegetal line		
C14:0	1.04	0.96	2.57	0.17
C16:0	28.23	24.87	30.75	12.57
C18:0	6.01	4.22	18.87	4.36
Saturated fatty acids	35.28	30.05	52.19	17.10
C16:1	2.21	1.15	3.16	1.21
C18:1	35.55	32.78	36.44	23.92
Monounsaturated fatty acids	37.76	33.93	39.60	25.13
C18:2	23.21	29.99	7.57	52.20
C18:3	1.57	2.47	0.64	5.57
Polyunsatured fat acids	24.78	32.46	8.21	57.77
Other	2.18	3.56	-	-

**Table 3:** Effect of ozonation on colour and drip loss of chicken breast meats

Rearing system	Standard line		Vegetal line		Significance			Pooled SE
		
	Control	Ozonated	Control	Ozonated	Feeding effect	O	F×O	
Colour								
L*	53.20	54.70	56.14	59.28	**	*	*	2.15
a*	0.57	0.97	−1.65	−1.01	*	*	*	0.14
b*	17.63	16.45	8.53	8.67	**	ns	*	3.47
Drip loss (%)	0.65	0.36	1.49	0.46	**	*	*	0.21

O, ozonation; F×O, interaction between feeding and ozonation; SE, standard error; ns, not significant.

* p<0.05,

** p<0 01.

**Table 4:** Effect of rearing system on lipid content (g/100 g of chicken breast meat) and fatty acid profile (g/100 g of total lipids)

Rearing system	Standard line	Vegetal line	Pooled SE
Lipid content (%)	1.53^a^	0.94^b^	0.25
Fatty acid profile (%)			
C14:0	1.51^b^	1.13^a^	0.20
C16:0	30.36	27.85	2.03
C18:0	11.59^a^	10.05^b^	1.01
Saturated fatty acids	43.46	39.03	2.35
C16:1	4.17^a^	3.06^b^	0.84
C18:1n-9	23.18	21.34	2.14
C18:1n-7	2.81	2.93	0.84
Monounsaturated fatty acids	30.16^a^	27.33^b^	1.68
C18:2n-6	17.95	22.14	1.54
C18:3n-3	0.60	0.85	0.18
C20:4n-6	4.19^a^	5.95^b^	0.48
Polyunsatured fat acids	22.74^a^	28.94^b^	2.11
Others	3.64^a^	4.70^b^	0.97

SE, standard error.

a,b Values in the same row followed by different letters differ significantly (p<0.05).

**Table 5:** Effect of ozonation on the lipid oxidative stability of chicken breast meats

Rearing system		Standard line		Vegetal line		Significance		
		
		Control	Ozonated	Control	Ozonated	Feeding effect	O	F×O
Thiobarbituric acid reactive substances (ppm)	External	1.22±0.25	3.52±0.50	1.59±0.22	5.13±0.39	**	**	*
	Internal	0.70±0.20	1.89±0.23	0.94±0.23	3.54±0.27	**	**	**

O, ozonation; F×O, interaction between feeding and ozonation.

* p<0.05,

** p<0.01.
